# Development and evaluation of an adenosine-to-inosine RNA editing-based prognostic model for survival prediction of bladder cancer patients

**DOI:** 10.1097/MD.0000000000033719

**Published:** 2023-05-12

**Authors:** Yin-Chao Tang, Chang-Shun Yang, Ming-Xing Liang, Yong Zhang, Yuan Liu, Shao-Hui Zou, Shu-Fan Shi

**Affiliations:** a Clinical Laboratory, The First People’s Hospital of Huaihua, Huaihua, Hunan, China.

**Keywords:** adenosine-to-inosine RNA editing, bladder cancer, nomogram, overall survival, prognosis

## Abstract

Adenosine-to-inosine RNA editing (ATIRE) is a common form of ribonucleic acid (RNA) editing, which has highlighted the importance of ATIRE in tumors. However, its role in bladder cancer (BLCA) remains poorly understood. To study ATIRE impact on BLCA patient prognosis, we obtained ATIRE, gene expression, and clinical data from the Cancer Genome Atlas (TCGA) database for 251 patients, randomly dividing them into training and testing groups. Univariate proportional hazards model (COX) regression identified prognosis-associated ATIRE loci, while the least absolute shrinkage and selection operator (LASSO) selected final loci to construct prognostic models and generate ATIRE scores. We developed a nomogram to predict BLCA patients’ overall survival (OS) and analyzed the effect of ATIRE editing levels on host gene expression. We also compared immune cell infiltration and drug treatment between patients with high and low ATIRE scores. The ATIRE prognostic prediction model was constructed using ten ATIRE loci that are closely associated with BLCA survival. Patients with high ATIRE scores showed significantly worse OS than those with low ATIRE scores. Furthermore, the nomogram, which incorporates the ATIRE score, can better predict the prognosis of patients. Multiple functional and pathway changes associated with immune responses, as well as significant differences in immune cell infiltration levels and response to drug therapy were observed between patients with high and low ATIRE scores. This study represented the first comprehensive analysis of the role of ATIRE events in BLCA patient prognosis and provided new insights into potential prognostic markers for BLCA research.

## 1. Introduction

Bladder cancer (BLCA) is a widespread form of cancer with consistently high incidence and mortality rates worldwide.^[1]^ However, the early symptoms of BLCA are often subtle and easily mistaken for other urinary tract conditions, leading to delayed diagnosis and difficulty in achieving complete resection.^[2,3]^ Despite advances in clinical management, predicting the prognosis of BLCA remains a challenge due to the complex molecular mechanisms and heterogeneous presentation of the disease.

In the past few years, high-throughput sequencing technology has revolutionized tumor research by providing a rapid and efficient way to analyze large-scale DNA, ribonucleic acid (RNA), and protein data. This has led to the discovery of many new prognostic markers and the development of novel treatment strategies.^[4,5]^ It is now well-established that gene mutations and abnormal expression play a crucial role in tumorigenesis and development.^[6,7]^ High-throughput sequencing technique can comprehensively detect gene mutation and abnormal expression, which provides a more accurate basis for tumor diagnosis and treatment.^[8]^ For instance, recent studies have identified numerous genes, including TP53, EGFR, and BRAF, that are associated with tumorigenesis and development using high-throughput sequencing techniques.^[7,9,10]^ Moreover, high-throughput sequencing technology has the potential to identify new prognostic markers. These markers play a crucial role in the treatment and management of tumors. By analyzing the genetic, RNA, and protein expression data in tumor tissues, new prognostic markers can be discovered, thereby improving the prognosis and survival rate of cancer patients.^[11]^ Currently, these markers are widely used in constructing tumor prognostic models. Nevertheless, there is still a shortage of prognostic prediction models related to RNA editing, particularly in BLCA research.

Adenosine-to-inosine RNA editing (ATIRE) is a type of RNA editing where adenosine (A) is converted to inosine (I) in RNA by adenosine deaminase. This process typically occurs in the structure of double-stranded RNA and is mediated by a specific adenosine deaminase (ADAR). ATIRE can impact the stability, structure, and function of RNA, ultimately influencing protein synthesis and biological function.^[12]^

In recent years, the importance of ATIRE in tumors has been increasingly recognized by a growing number of studies. For instance, ATIRE has been found to play a role in tumor cell proliferation, metastasis, and treatment response.^[13]^ Specifically, it can affect the post-transcriptional regulation of tumor cells, including RNA splicing, stability, and translation, which can impact protein synthesis and biological function.^[14]^ Furthermore, evidence suggests that ATIRE can influence the immune response in tumor cells, which in turn can affect tumor progression and response to treatment.^[15]^ Given its multifaceted impact on tumorigenesis and progression, ATIRE has emerged as a promising area of research. However, to date, there have been no studies investigating ATIRE-based prognostic prediction models for BLCA, and its role in BLCA prognosis remains largely unexplored.

In this study, we sought to develop an ATIRE prediction model for BLCA patients by analyzing the ATIRE profile and clinical data from The Cancer Genome Atlas (TCGA) database. Our goal was to identify ATIRE loci that are associated with overall survival (OS) and construct a nomogram that predicts OS in BLCA based on ATIRE scores and clinicopathological features. Additionally, we aimed to explore differences in the immune microenvironment and treatment response among patients with different ATIRE scores. Through these efforts, our study offered a more comprehensive assessment of the potential role and mechanisms of ATIRE loci in BLCA prognosis.

## 2. Materials and methods

### 2.1. Data acquisition and processing

TCGA-BLCA ATIRE data were obtained from the TCGA_RNA editing dataset uploaded by Han L et al on the Synapse data storage website (https://www.synapse.org/#!Synapse:syn2374375).^[16]^ Transcriptomic data in TPM format and clinical information for the corresponding samples were obtained from the TCGA database (https://portal.gdc.cancer.gov/). Progression-free survival (PFS) information and Stemness Scores for BLCA patients were obtained from UCSC Xena (https://xena.ucsc.edu/).^[17]^ The ATIRE dataset for BLCA contains 271 samples containing 19 normal tissues and 252 BLCA tissues. After removing BLCA samples with incomplete prognostic information and editing loci containing 50% of the missing values, 251 BLCA samples were used for subsequent analysis.

### 2.2. Univariate proportional hazards model (COX) regression analysis and model construction

The obtained ATIRE data were screened for BLCA prognosis-related ATIRE loci by univariate COX regression analysis using the R language “survival” package. Then 251 BLCA tissues were randomly divided into a training group and a testing group according to 1:1 using the R language “caret” package. In the training group, the prognosis-related RNA editing loci data were modeled using the “glmnet” package for the least absolute shrinkage and selection operator (LASSO) regression analysis, and lambda.1se was chosen for the λ value. Patients in the training group, testing group, and entire group were divided into high and low ATIRE score patients according to the median ATIRE score of the training group. The time-dependent receiver operating characteristic curve (ROC) was plotted with the “timeROC” package. The independent prognostic analysis of the model was based on univariate COX regression and multivariate COX regression analysis of the obtained model and other clinical characteristics of the patients (Age, Gender, Stage, T stage, Race, and Therapy).

### 2.3. Development and evaluation of nomogram based on ATIRE

The “rms” package was used to create the OS nomogram. The clinical information included in the nomogram along with the ATIRE score are Age, Gender, Stage, and TNM stage. To validate the established nomogram, total scores were calculated for all patients based on the established nomogram, followed by CoxPH regression and assessment of Harrell C-index and calibration. A decision curve analysis (DCA) was conducted to determine the clinical utility of different prognostic models by quantifying the net benefit at different threshold probabilities using the R language “ggDCA” package. We also performed a time-dependent ROC analysis comparing the efficacy of various factors in predicting prognosis.

### 2.4. Transcriptome data analysis and enrichment analysis

To understand the possible regulatory mechanism of ATIRE loci in BLCA, we used the Spearman rank test to analyze the correlation between ATIRE level and host gene expression, and also analyzed the correlation between ATIRE level and ADAR family gene expression (ADAR, ADARB1, and ADARB2). The differentially expressed genes between high and low ATIRE score groups were analyzed by using the R language “limma” package, and the screening conditions were | logFC | > 1 and FDR < 0.05. The enrichment analysis was carried out by the “clusterProfiler” package, and the screening condition of the enrichment result was *P* < .05.

### 2.5. Differences of immune cell infiltration and treatment response in patients with high and low ATIRE score

BLCA immune cell infiltration data were obtained from TIMER 2.0 (http://timer.cistrome.org/).^[18]^ The dataset also contains the results of the immuno-infiltration level assessment by 7 software packages (TIMER, XCELL, QUANTISEQ, MCPCOUNTER, EPIC, CIBERSORT-ABS, and CIBERSORT), which considerably increases the reliability of the results. The correlation between ATIRE score and immune cell infiltration was analyzed by the Spearman rank test. The immunotherapy score was completed by Tumor Immune Dysfunction and Exclusion (TIDE) algorithm (http://tide.dfci.harvard.edu/).^[19,20]^ IC50 analysis of BLCA chemotherapeutic drugs was performed with “pRRophetic” package.

### 2.6. Statistical analysis

Demographic and baseline characteristics of the different subgroups in this study were compared using the chi-square test. OS and PFS survival analyses were performed using the Kaplan–Meier method. The Wilcoxon test was used for comparison between the 2 groups for continuous variables. All statistical analyses for this study were completed in R software (4.1.3) and *P *< .05 was considered statistically significant.

## 3. Results

### 3.1. Baseline clinicopathological features of BLCA patients

After removing 50% of missing ATIRE loci from the BLCA_ATIRE dataset and samples with incomplete prognostic information, a total of 251 BLCA samples were obtained. We randomly divided these samples into 2 groups according to 1:1 and obtained 127 patients in the training group and 124 patients in the testing group. The clinicopathological characteristics of the training, testing, and entire groups are shown in Table 1. There were no significant differences between the 3 groups in Age, Gender, Grade, T, N, M, Race, Therapy methods, and Survival status, indicating that the groupings were reasonable and unbiased.

**Table 1:** Frequency distribution of demographic and clinicopathological characteristics of BLCA patients.

Covariates	Type	Total (entire)	Testing	Training	*P* value
Age	<=65	95 (37.85%)	43 (34.68%)	52 (40.94%)	.3716
	>65	156 (62.15%)	81 (65.32%)	75 (59.06%)	
Gender	Female	60 (23.9%)	28 (22.58%)	32 (25.2%)	.7355
	Male	191 (76.1%)	96 (77.42%)	95 (74.8%)	
Grade	High grade	230 (91.63%)	112 (90.32%)	118 (92.91%)	.9274
	Low grade	19 (7.57%)	10 (8.06%)	9 (7.09%)	
	Unknown	2 (0.8%)	2 (1.61%)	0 (0%)	
Stage	Stage I–II	80 (31.87%)	42 (33.87%)	38 (29.92%)	.5318
	Stage III–IV	169 (67.33%)	80 (64.52%)	89 (70.08%)	
	Unknown	2 (0.8%)	2 (1.61%)	0 (0%)	
T	T1–2	73 (29.08%)	38 (30.65%)	35 (27.56%)	.7423
	T3–4	156 (62.15%)	76 (61.29%)	80 (62.99%)	
	Unknown	22 (8.76%)	10 (8.06%)	12 (9.45%)	
M	M0	126 (50.2%)	64 (51.61%)	62 (48.82%)	.4997
	M1	7 (2.79%)	5 (4.03%)	2 (1.57%)	
	Unknown	118 (47.01%)	55 (44.35%)	63 (49.61%)	
N	N0–1	178 (70.92%)	90 (72.58%)	88 (69.29%)	.9714
	N2–3	53 (21.12%)	26 (20.97%)	27 (21.26%)	
	Unknown	20 (7.97%)	8 (6.45%)	12 (9.45%)	
Race	Asian	30 (11.95%)	17 (13.71%)	13 (10.24%)	.5004
	Black or African American	11 (4.38%)	4 (3.23%)	7 (5.51%)	
	Unknown	35 (13.94%)	17 (13.71%)	18 (14.17%)	
	White	175 (69.72%)	86 (69.35%)	89 (70.08%)	
Therapy methods	Pharmaceutical therapy	100 (39.84%)	47 (37.9%)	53 (41.73%)	.5782
	Radiation therapy	116 (46.22%)	60 (48.39%)	56 (44.09%)	
	Unknown	35 (13.94%)	17 (13.71%)	18 (14.17%)	
Survival status	Alive	148 (58.96%)	65 (52.42%)	83 (65.35%)	.0506
	Dead	103 (41.04%)	59 (47.58%)	44 (34.65%)	

BLCA = bladder cancer.

### 3.2. Prognosis-related ATIRE loci screening and prognostic model construction

**Figure 1.**
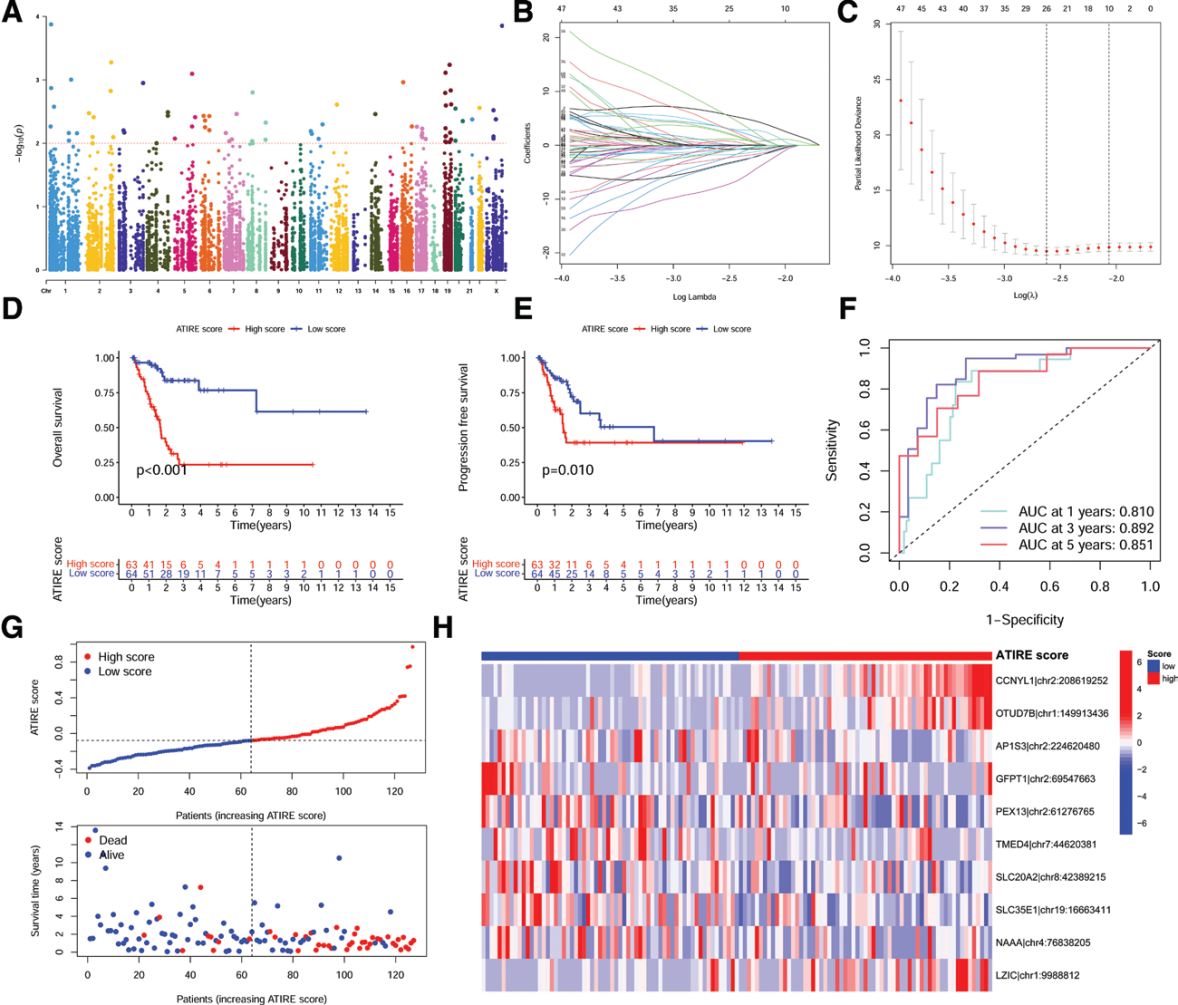
Prognosis-related ATIRE loci screening and prognostic model construction. (A) Univariate COX regression screening prognosis related ATIRE loci. (B) LASSO regression: each curve represents the changing track of each independent variable coefficient, with the change of λ value, the later the coefficient is compressed to 0, the more important the variable. (C) LASSO regression: the curve of Partial-likelihood deviance changing with Log (λ), the smaller the value, the better the model fitting. (D) Overall prognostic survival analysis of high and low ATIRE score groups. (E) PFS analysis of high and low ATIRE score groups. (F) ROC curves for prognostic models predicting 1-, 3-, 5-yr prognosis. (G) Changes in survival status of patients with increasing ATIRE scores. (H) Heat map of the editing level of ATIRE loci. ATIRE = adenosine-to-inosine RNA editing, COX = proportional hazards model, LASSO = the least absolute shrinkage and selection operator, PFS = progression-free survival, RNA = ribonucleic acid, ROC = receiver operating characteristic curve.

We used univariate COX regression analysis to screen prognostic-related ATIRE loci based on 251 BLCA samples, and a total of 77 ATIRE loci were obtained (Fig. 1A; Supplemental Table S1, http://links.lww.com/MD/I945, which demonstrates the univariate COX regression analysis results of ATIRE loci in BLCA). We used LASSO regression in the training group to construct a prognostic model consisting of 10 ATIRE loci (Fig. 1B and C), the editing levels of which were all significantly associated with the prognosis of BLCA patients (Supplemental Fig. S1, http://links.lww.com/MD/I946, which shows the prognostic analysis of the selected ATIRE loci in the model). The risk score calculated by the model is defined in this study as ATIRE score = (3.09 × CCNYL1|chr2:208619252) + (1.11 × OTUD7B|chr1:149913436) + (−0.24 × AP1S3|chr2:224620480) + (−1.81 × GFPT1|chr2:69547663) + (−0.10 × PEX13|chr2:61276765) + (−0.72 × TMED4|chr7:44620381) + (−0.43 × SLC20A2|chr8:42389215) + (−0.79 × SLC35E1|chr19:16663411) + (−1.00 × NAAA|chr4:76838205) + (1.49 × LZIC|chr1:9988812). In the training group, we use this formula to calculate the ATIRE score of each sample, and use the median value of ATIRE score to divide BLCA patients into high ATIRE score group and low ATIRE score group, and then compare the OS between the 2 groups. We find that the survival time of the high ATIRE score group was significantly lower than that of the low ATIRE score group (Fig. 1D; *P <* .001). Similar results were obtained in the PFS (Fig. 1E; *P* = .010). The 1-, 3- and 5-year AUCs for OS prediction were 0.810, 0.892, and 0.851 respectively, indicating good predictive efficacy (Fig. 1F). The results of the prognostic status analysis showed that the number of deaths increased with the increase of the ATIRE score (Fig. 1G). In addition, the editing level heat map of ATIRE loci showed that the editing level of ATIRE loci with a positive regression coefficient in the high ATIRE score group was higher than that in the low ATIRE score group, while the editing level of negative regression coefficient in high ATIRE score group was lower than that in low ATIRE score group (Fig. 1H).

### 3.3. Verification of the prognostic model in the testing group and the entire group

**Figure 2.**
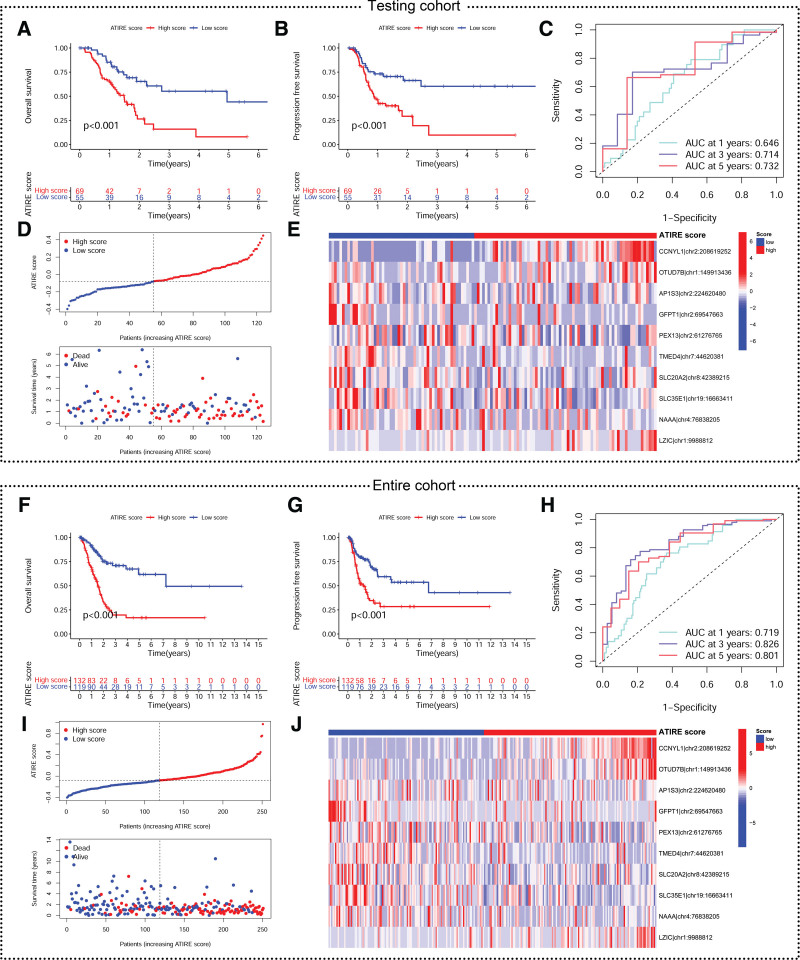
Prognostic model validation in the testing and entire groups. (A) Overall survival analysis of patients with high and low ATIRE scores in the testing group. (B) Progression-free survival analysis of patients with high and low ATIRE scores in the testing group. (C) ROC curve for predicting prognosis of patients in the testing group. (D) Survival status change of patients in the testing group with increasing ATIRE scores. (E) Heatmap showing the editing levels of ATIRE loci in the testing group. (F) Overall survival analysis of patients with high and low ATIRE scores in the entire group. (G) Progression-free survival analysis of patients with high and low ATIRE scores in the entire group. (H) ROC curve for predicting prognosis of patients in the entire group. (I) Survival status change of patients in the entire group with increasing ATIRE scores. (J) Heatmap showing the editing levels of ATIRE loci in the entire group. ATIRE = adenosine-to-inosine RNA editing, ROC = receiver operating characteristic curve.

We validated the prognosis model in the testing group and the entire group respectively. The prognostic model obtained in the training group was used to calculate the ATIRE score for each BLCA patient in the testing and entire groups respectively, and then the median ATIRE score in the training group was used to classify the patients in the testing and entire groups as high ATIRE score and low ATIRE score patients respectively. Analysis in the testing group showed that patients with high ATIRE scores had significantly worse OS and PFS than patients with low ATIRE scores (Fig. 2A and B; *P* < .001). The predicted AUC values for OS at 1-, 3-, and 5-years were 0.646, 0.714, and 0.732, respectively (Fig. 2C), and the heat map of patient survival status and RNA editing levels were similar to the training group (Fig. 2D and E). Similar results were obtained in the entire group, with OS and PFS significantly worse in patients with high ATIRE scores than in those with low ATIRE scores (Fig. 2F and G; *P* < .001), with predicted AUC values of 0.719, 0.826, and 0.801 for OS at 1-, 3-, and 5-years, respectively (Fig. 2H), and patient survival status and RNA editing heat map were similar to the training group as well (Fig. 2I and J).

### 3.4. Independent prognostic analysis of the ATIRE score

**Figure 3.**
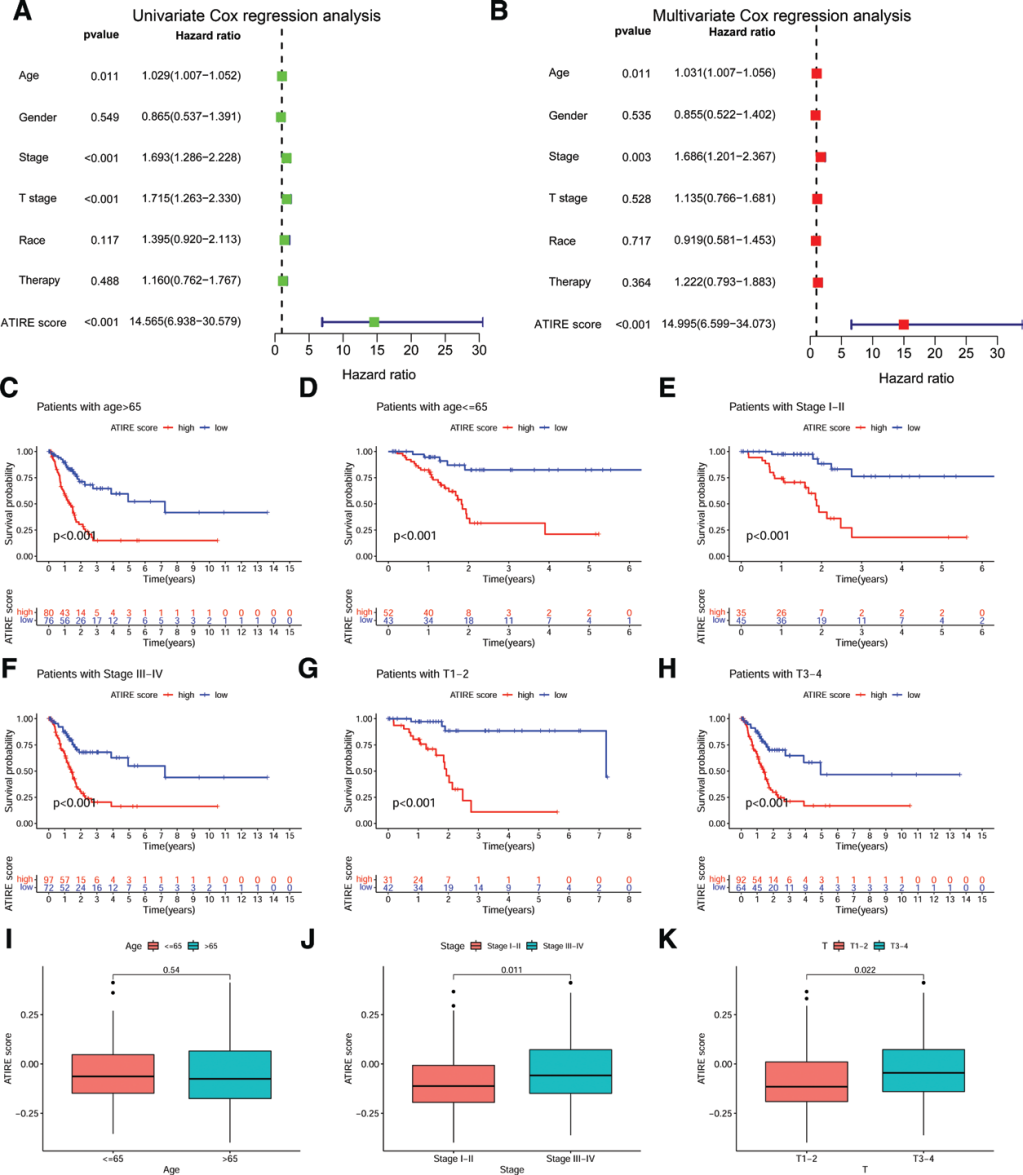
Independent prognostic analysis of ATIRE score and correlation analysis with clinical features. (A) Univariate Cox regression analysis. (B) Multivariate Cox regression analysis. (C–H) Stratified analysis of ATIRE score. (I–J) Correlation analysis of ATIRE score with Age, stage, and T. ATIRE = adenosine-to-inosine RNA editing, COX = proportional hazards model.

To investigate whether the ATIRE score is independent of other clinical features in predicting the prognosis of BLCA patients, we performed univariate and multivariate Cox regression analyses of the ATIRE score and other clinical features (Age, Gender, Stage, T stage, Race, and Therapy) separately. As shown in Figure 3A and B, the ATIRE score was significantly associated with patient prognosis in both univariate and multivariate Cox regression analyses (*P* < .001). Stratified analyses of clinical features with *P* < .05 in univariate Cox regression analysis showed that patients with high ATIRE scores had significantly lower survival than those with low ATIRE scores in Age > 65, Age ≤ 65, Stage I to II, Stage III to IV, T1–2, and T3–4 patients (Fig. 3C–H; *P* < .001). Additionally, there was no difference in ATIRE score between Age > 65 and Age ≤ 65 patients (Fig. 3I), while ATIRE score was significantly higher in Stage III to IV than in Stage I to II patients (Fig. 3J; *P *= .011) and T3–4 than in T1–2 patients (Fig. 3K; *P* = .022). These results indicate that the ATIRE score could serve as an independent prognostic factor for BLCA patients and is associated with tumor malignancy.

### 3.5. Prognostic nomogram development and evaluation in BLCA patients

**Figure 4.**
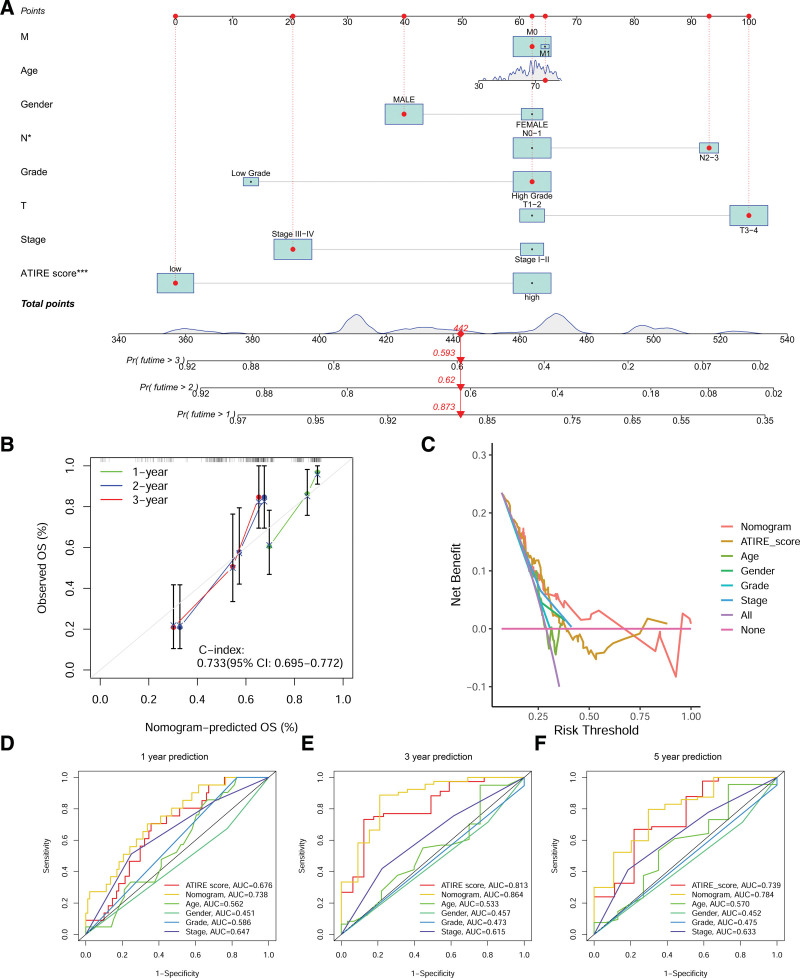
Construction and evaluation of prognostic nomogram for BLCA patients. (A) Nomogram. (B) Calibration curve for 1-, 3-, and 5-yr prognostic predictions. (C) Decision curve analysis (DCA) curve. (D–F) ROC curves for multiple indicators used for 1-, 3-, and 5-yr prognostic predictions in BLCA patients. BLCA = bladder cancer, ROC = receiver operating characteristic curve.

We also developed a nomogram for predicting the prognosis of BLCA patients based on the constructed prognostic prediction model (Fig. 4A). The calibration curves for 1-, 3-, and 5-year prognosis prediction are shown in Figure 4B, indicating that the nomogram has ideal predictive performance. The DCA results showed that the nomogram had a better net benefit than other prognostic prediction models (Fig. 4C), and the results of ROC curve analysis were consistent with those of DCA (Fig. 4D–F). These results further demonstrate that the prognostic prediction model and nomogram we constructed have good prognostic prediction values and are superior to other clinical features.

### 3.6. Correlation between ATIRE loci editing levels and host gene expression

**Figure 5.**
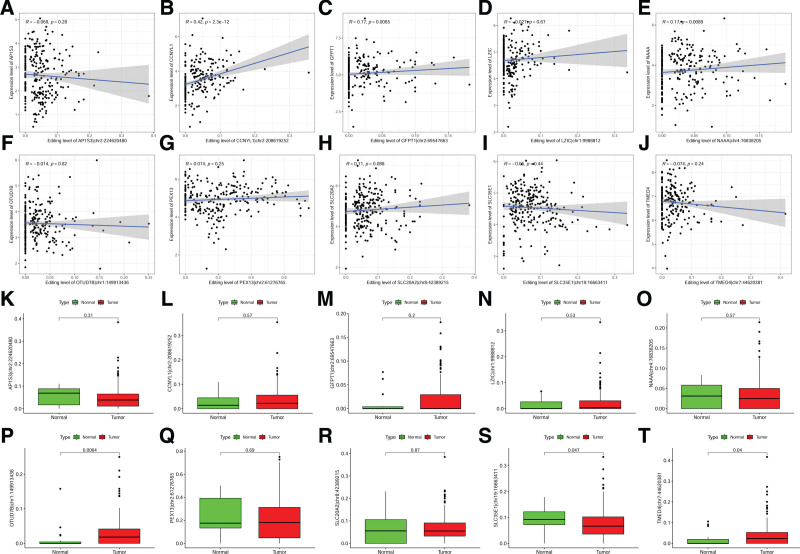
Analysis of ATIRE loci editing levels in the model. (A–J) Correlation analysis between ATIRE loci and host gene expression. (K–T) Comparison of RNA editing levels between normal and BLCA tissues. ATIRE = adenosine-to-inosine RNA editing, BLCA = bladder cancer, RNA = ribonucleic acid.

ATIRE can participate in regulating tumor development by affecting host gene expression.^[21]^ As shown in Figure 5A–J, there was no significant correlation between AP1S3|chr2:224620480 and AP1S3 expression (*R* = −0.069, *P *= .28), while CCNYL1|chr2:208619252 showed a significant positive correlation with CCNYL1 expression (*R* = 0.42, *P* = 2.3e − 12); GFPT1|chr2:69547663 showed a significant positive correlation with GFPT1 expression (*R* = 0.17, *P* = .0065); LZIC|chr1:9988812 showed no significant correlation with LZIC expression (*R* = − 0.027, *P* = .67); NAAA|chr4:76838205 showed a significant positive correlation with NAAA expression (*R* = 0.17, *P* = .0089); OTUD7B|chr1:149913436 showed no significant correlation with OTUD7B expression (*R* = −0.014, *P* = .82); PEX13|chr2:61276765 showed no significant correlation with PEX13 expression (*R* = 0.074, *P* = .25); SLC20A2|chr8:42389215 showed no significant correlation with SLC20A2 expression (*R* = 0.11, *P* = .088); SLC35E1|chr19:16663411 showed no significant correlation with SLC35E1 expression (*R* = −0.05, *P* = .44); TMED4|chr7:44620381 showed no significant correlation with TMED4 expression (*R* = −0.074, *P* = .24). Additionally, we compared the editing levels of these loci between normal samples and BLCA (Fig. 5K–T) and found that OTUD7B|chr1:149913436 (*P *= .0064) and OTUD7B|chr1:149913436 (*P* = .04) had significantly higher editing levels in tumors than in normal tissues, while only SLC35E1|chr19:16663411 (*P* = .047) showed significantly higher editing levels in normal tissues than in tumors. There was no significant difference in editing levels for the remaining loci.

### 3.7. The correlation between ATIRE loci editing levels and ADAR family gene expression

**Figure 6.**
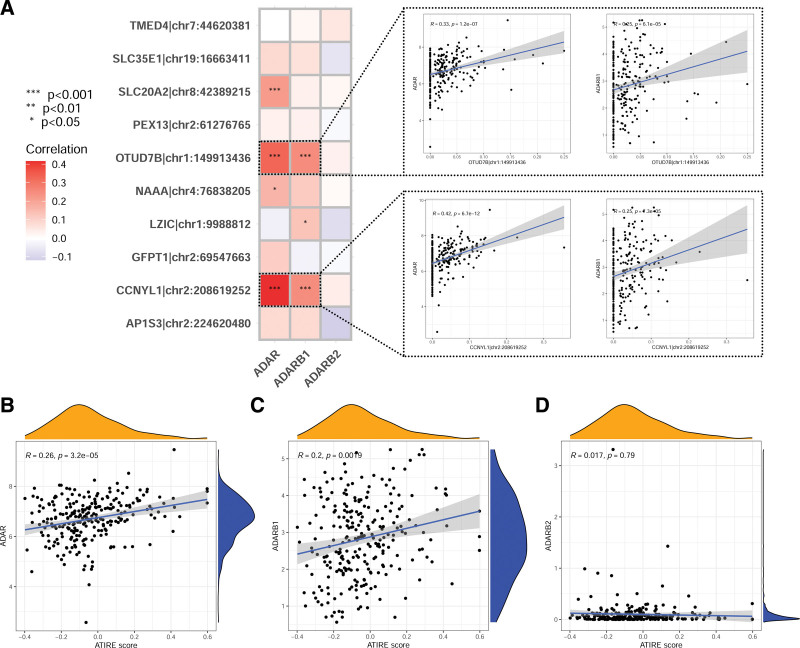
Correlation analysis of ADAR family gene expression with RNA editing levels and ATIRE score. (A) Correlation between ATIRE locus editing levels and expression of ADAR family genes (ADAR, ADARB1, and ADARB2). (B and C) Correlation between ATIRE score and expression of ADAR family genes. ADAR = adenosine deaminase, ATIRE = adenosine-to-inosine RNA editing, RNA = ribonucleic acid.

We compared the correlation between the editing levels of the ATIRE loci in the model and the expression of ADAR family genes (ADAR, ADARB1, and ADARB2) separately.^[12]^ As shown in Figure 6A, OTUD7B|chr1:149913436 and CCNYL1|chr2:208619252 were significantly positively correlated with the expression of ADAR and ADARB1 (*P* < .001); SLC20A2|chr8:42389215 was significantly positively correlated with ADAR expression (*P* < .001); NAAA|chr4:76838205 was significantly positively correlated with ADAR expression (*P* < .05); LZIC|chr1:9988812 was significantly positively correlated with ADARB1 expression (*P *< .05). It is worth noting that ADARB2 was not significantly correlated with the editing levels of any of the loci. We also analyzed the correlation between the ATIRE score calculated based on the editing levels of these loci in the model and the expression of ADAR, ADARB1, and ADARB2, and found that the ATIRE score was significantly positively correlated with ADAR and ADARB1 (Fig. 6B and C), while there was no significant correlation with ADARB2 (Fig. 6D).

### 3.8. Differential genes and related biological pathways between high and low ATIRE score patients

**Figure 7.**
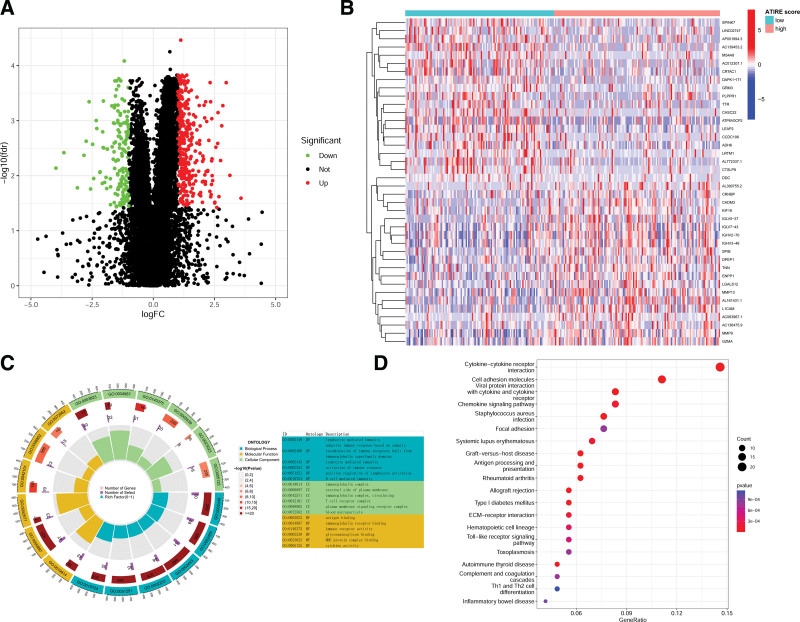
Differential genes and related biological pathways between high and low ATIRE score patients. (A) Volcano plot of differentially expressed genes (red dots represent upregulated genes, green dots represent downregulated genes, and black dots represent genes with no significant difference). (B) Heatmap of the top 20 upregulated and downregulated genes. (C) Results of GO enrichment analysis. (D) Results of KEGG enrichment analysis. ATIRE = adenosine-to-inosine RNA editing, GO = Gene Ontology, KEGG = Kyoto Encyclopedia of Genes and Genomes.

Under the condition of |logFC| > 1 and FDR < 0.05, 493 differentially expressed genes were screened out (Fig. 7A), and the top 20 up-regulated and down-regulated genes are shown in the heatmap in Figure 7B. Gene Ontology enrichment analysis results showed that these 493 genes were mainly enriched in immune-related terms (Fig. 7C). Kyoto Encyclopedia of Genes and Genomes enrichment analysis showed that these genes were mainly enriched in some inflammatory response and immune-related pathways (Fig. 7D).

### 3.9. The relationship between ATIRE score and patient treatment

**Figure 8.**
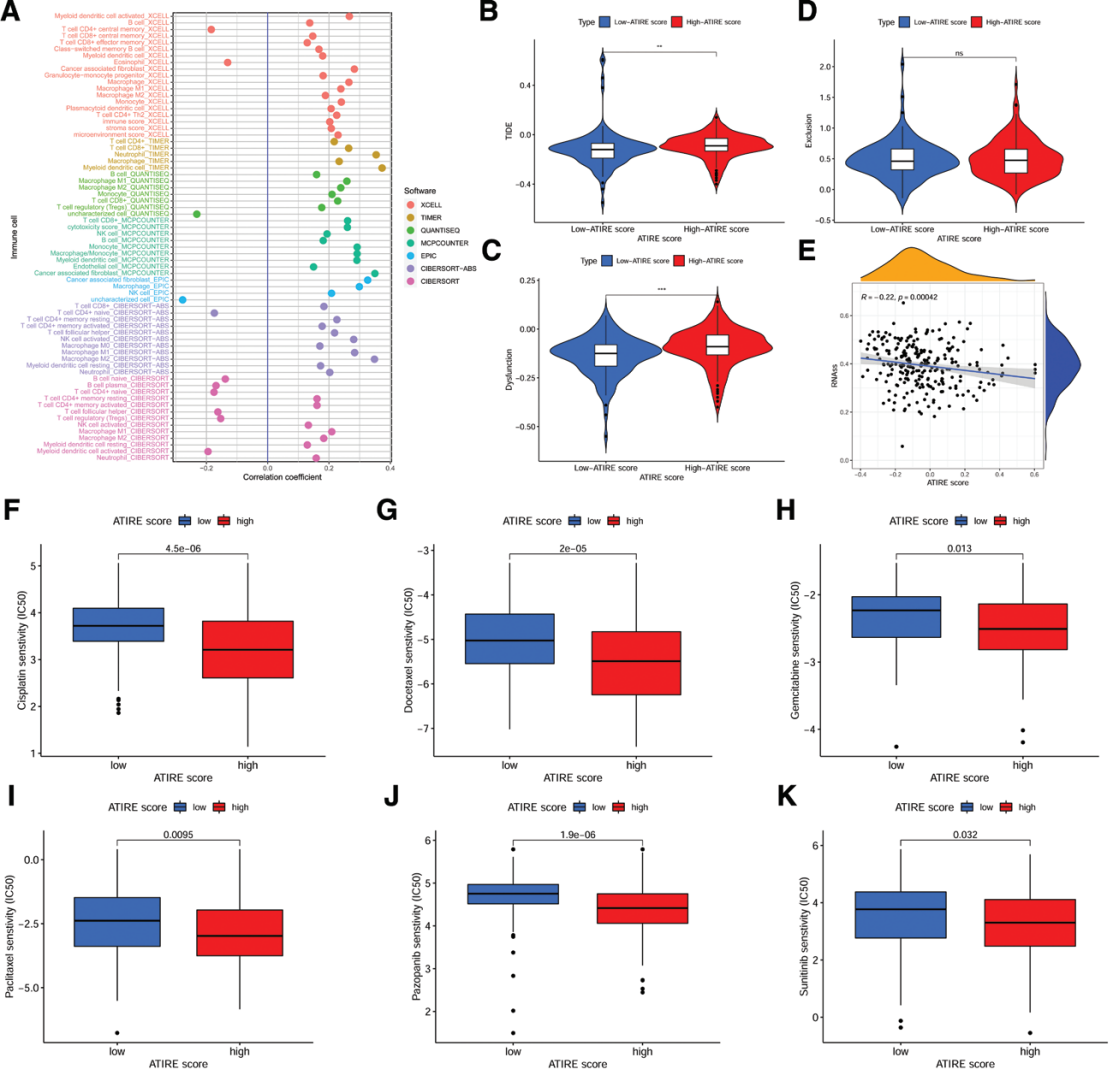
The relationship between ATIRE score and patient treatment. (A) Correlation analysis between ATIRE score and BLCA immune cell infiltration levels. (B) Comparison of TIDE immune treatment scores between high and low ATIRE score groups. (C) Comparison of Dysfunction immune treatment scores between high and low ATIRE score groups. (D) Comparison of Exclusion immune treatment scores between high and low ATIRE score groups. (E) Correlation analysis between ATIRE score and RNAss. (F–K) Comparison of IC50 values of commonly used chemotherapy drugs between high and low ATIRE score groups. ATIRE = adenosine-to-inosine RNA editing, BLCA = bladder cancer, TIDE = tumor immune dysfunction and exclusion, RNA = ribonucleic acid.

The above results suggested that the ATIRE score was related to patient immune function, which prompted us to further analyze the relationship between ATIRE score and patient immune microenvironment and immune therapy, providing a reference for drug treatment of patients with different ATIRE scores. The analysis of the immune microenvironment showed that the ATIRE score was positively correlated with the infiltration level of most immune cells (Fig. 8A). The immune therapy score TIDE was significantly higher in the high ATIRE score group than in the low ATIRE score group (Fig. 8B; *P* < .01), indicating a higher likelihood of immune evasion in high-risk patients and poor efficacy of immune checkpoint blockade therapy. The analysis of immune dysfunction was consistent with TIDE, with the high ATIRE score group significantly higher than the low ATIRE score group (Fig. 8C, *P* < .001), while there was no significant difference in the immune exclusion score between the 2 groups (Fig. 8D).

We also unexpectedly found a significant negative correlation between the ATIRE score and the BLCA stemness score (RNAss) (Fig. 8E; *R* = −0.22, *P* < .001). Patients with stronger tumor stemness are known to be resistant to chemotherapy drugs, suggesting that patients in the high ATIRE score group may have better chemotherapy. We then compared the IC50 of commonly used chemotherapy drugs, including cisplatin, docetaxel, gemcitabine, paclitaxel, pazopanib, and sunitinib, between the 2 groups of patients. The results are shown in Figure 8F–K, where the IC50 of these drugs in the high ATIRE score group was significantly lower than that in the low ATIRE score group, indicating that the high ATIRE score group had better chemotherapy drug sensitivity, consistent with the analysis results of RNAss.

## 4. Discussion

Building a reliable prognostic model is crucial for the treatment and management of BLCA patients. In recent years, increasing evidence has shown that ATIRE plays an important role in the occurrence and development of various cancers, including BLCA.^[22]^ However, no study has yet constructed a BLCA prognostic model based on ATIRE loci. By analyzing the ATIRE profile in TCGA of BLCA, we can identify ATIRE loci associated with overall patient survival and develop a prognostic model for predicting the survival of BLCA patients. This will help guide patient treatment and management, and improve the survival rate and quality of life of BLCA patients. In previous studies, many teams have been dedicated to building prognostic prediction models for BLCA to guide clinical treatment and management. However, existing prognostic prediction models are usually based on a small number of clinical and pathological factors and molecular biomarkers, which limits their accuracy and reliability.^[23–25]^ In terms of reliability, ATIRE editing levels are more stable than gene expression quantification. In addition, the role of ATIRE in BLCA prognosis is still not fully understood. Therefore, it is necessary to explore new predictive models and construct an ATIRE-based prognostic model for BLCA.

Our study constructed a prognostic risk model consisting of 10 ATIRE loci and calculated the ATIRE score for each patient to evaluate their OS and RFS. Our analysis results also showed that the ATIRE score could serve as an independent prognostic factor for BLCA patients and was related to the malignancy degree of tumors. The nomogram constructed by combining the ATIRE score with clinical features had a better predictive performance for OS prediction in BLCA patients and outperformed other included clinical features. To our knowledge, this is the first study using ATIRE events to predict survival in BLCA patients.

Given the importance of ATIRE in tumors, we identified 10 ATIRE loci as the optimal prognostic factors for BLCA using the univariate Cox method combined with LASSO regression. Among these relevant genes, NAAA encodes a highly similar N-acyl ethanolamine hydrolase (NAEs) to acid neuraminidase, which has been reported to play an important role in BLCA cell migration and induction of tumor cell death and may be a therapeutic target for relevant inhibitors.^[26]^ The remaining genes are first reported to be associated with the prognosis of BLCA. However, these genes have also been reported to be associated with the occurrence and development of other types of tumors. For example, OTUD7B has been reported to play a critical role in activating mTORC2/AKT signaling and promoting lung cancer,^[27]^ and its abnormal expression is also associated with the progression of breast cancer,^[28]^ pancreatic cancer,^[29]^ and liver cancer.^[30]^ High expression of AP1S3 is closely related to the proliferation, invasion, and migration of glioma cells.^[31]^ High expression of GFPT1 is associated with the proliferation of cervical cancer cells.^[32]^ A recent study team found that the PEX13 gene was significantly upregulated in both single-cell and bulk RNA sequencing data, and the expression level of PEX13 was associated with tumor stemness. PEX13 may be an important tumor stemness marker in tumors and can be used as a biomarker to predict the immunotherapy responsiveness of tumors.^[33]^ TMED4 is a transmembrane protein that plays an important role in the intracellular membrane system. Recent studies have shown that TMED4 also plays an important role in tumor occurrence and development. For example, studies have shown that TMED4 is highly expressed in lung cancer and is associated with tumor growth and metastasis.^[34]^ SLC20A2 is a member of the family of inorganic phosphate transporters. Studies have found that SLC20A2 is associated with perineural invasion in stage II colorectal cancer.^[35]^ SLC35E1 belongs to the solute carrier family 35. This gene has been reported to be associated with the recurrence of papillary thyroid carcinoma.^[36]^ LZIC has also been reported to be associated with tumor cell proliferation, invasion, metastasis, angiogenesis, apoptosis, and immune evasion.^[37,38]^ In this study, we reported for the first time the role of these genes in BLCA prognosis from the perspective of ATIRE, which is conducive to the discovery of more BLCA prognostic markers in the future.

The mechanism by which these loci in the model affect the prognosis of BLCA patients is still unclear. It has been reported that ATIRE can affect gene expression by changing RNA sequence, thereby altering protein sequence or function, and affecting alternative splicing, RNA stability, and miRNA binding.^[12,39]^ We found significant positive correlations between chr2:208619252 and CCNYL1 expression, chr2:69547663 and GFPT1 expression, and chr4:76838205 and NAAA expression. The expression of the key editing enzymes of ATIRE, the ADAR family genes ADAR and ADARB1, is significantly positively correlated with the editing levels of these loci, consistent with the theoretical basis that the expression levels of ADAR and ADARB1 are highly correlated with the levels of ATIRE.^[12]^ In addition, the lack of significant correlation between the editing levels of some loci and host gene expression does not necessarily mean that these loci are unrelated to host gene expression, as mRNA levels do not represent protein levels regulated after transcription, and confirmation of protein levels through experiments is necessary. Further research is needed to determine whether these loci may affect patient prognosis by regulating host gene expression levels. In addition, we also found differences in editing levels of some loci between tumor and normal tissues, such as chr1:149913436 and chr19:16663411, indicating that these loci may be involved in BLCA development.

We utilized the prognostic model composed of these 10 loci to calculate the ATIRE score for each patient. We compared the differential genes and related biological pathways between patients with high and low ATIRE scores and found significant changes in immune response-related functions and pathways. Consistent with previous reports, ATIRE can regulate the biological processes of immune cells, and RNA modifications can participate in the pathogenesis of immune-related diseases such as cancer, infection, inflammation, and autoimmune diseases.^[40–42]^ Immune microenvironment analysis results showed that the ATIRE score was positively correlated with the infiltration level of most immune cells. Furthermore, we found that the immune therapy score TIDE and the immune dysfunction score were significantly higher in the high ATIRE score group than in the low ATIRE score group, indicating that patients in the high-risk group had a poorer response to immune checkpoint blockade therapy. This suggests that one of the reasons for the significant difference in prognosis between patients with high and low ATIRE scores may be the differences in tumor immune responses caused by differential RNA modifications.

To further demonstrate the difference in treatment between the high and low ATIRE score groups, we compared the IC50 of commonly used chemotherapy drugs, including cisplatin, docetaxel, gemcitabine, paclitaxel, pazopanib, and sunitinib, between the 2 patient groups. The IC50 of the high ATIRE score group was significantly lower than that of the low ATIRE score group, indicating that the high ATIRE score group has better sensitivity to chemotherapy drugs and that the ATIRE score can be further used for drug selection.

However, this study has some limitations. Firstly, we only analyzed patients from the TCGA cohort and lack independent external data to validate this ATIRE prognostic model. Secondly, the prognostic nomogram we constructed included relatively few factors due to the limited patient information provided by TCGA. Finally, protein-level experiments are needed to further verify the impact of ATIRE loci editing levels on host protein expression.

In conclusion, we have constructed for the first time an ATIRE-based prognostic risk score model for BLCA patients. The nomogram incorporating the ATIRE score and clinical-pathological features has good predictive performance for the OS of BLCA patients. Our results also provided new clues for the study of prognostic biomarkers for BLCA.

## Author contributions

**Conceptualization:** Yin-Chao Tang, Chang-Shun Yang, Shu-Fan Shi.

**Data curation:** Yin-Chao Tang, Ming-Xing Liang, Shao-Hui Zou, Shu-Fan Shi.

**Formal analysis:** Ming-Xing Liang, Shu-Fan Shi.

**Funding acquisition:** Shu-Fan Shi.

**Investigation:** Yong Zhang.

**Methodology:** Chang-Shun Yang, Yong Zhang.

**Project administration:** Yin-Chao Tang.

**Resources:** Yin-Chao Tang, Shao-Hui Zou.

**Software:** Yin-Chao Tang, Shu-Fan Shi.

**Supervision:** Shao-Hui Zou, Shu-Fan Shi.

**Validation:** Yuan Liu.

**Visualization:** Yuan Liu, Shu-Fan Shi.

**Writing – original draft:** Yin-Chao Tang, Chang-Shun Yang, Shu-Fan Shi.

**Writing – review & editing:** Yin-Chao Tang, Shu-Fan Shi.

## Supplementary Material




