# Supplementary figures and images for: Development and evaluation of an adenosine-to-inosine RNA editing-based prognostic model for survival prediction of bladder cancer patients
Source: Medicine (Baltimore). 2023 May 12;102(19):e33719. doi: 10.1097/MD.0000000000033719 (PMC10174396; doi:10.1097/MD.0000000000033719)

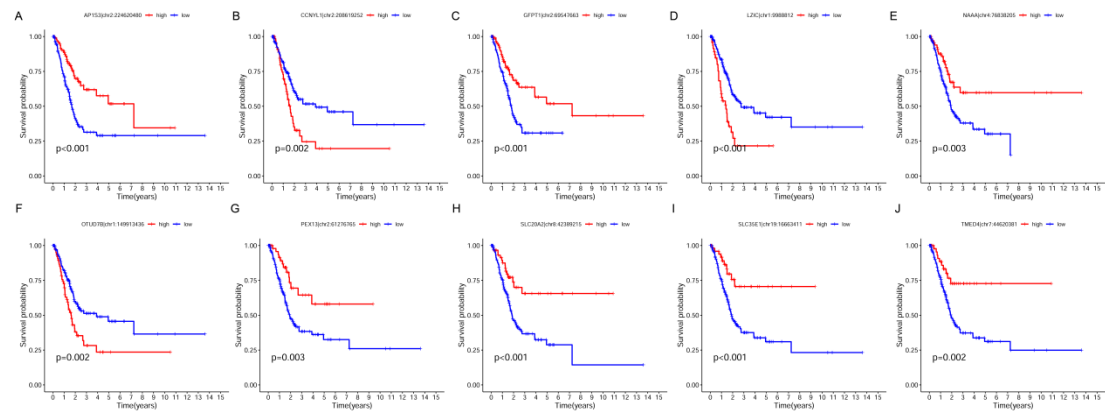

**FIGURE S1**

Prognostic analysis of the selected ATIRE loci in the model

Supplement: Supplementary file 2 [file medi-102-e33719-s002.pdf]
